# Family medicine in times of ‘COVID-19’: A generalists' voice

**DOI:** 10.1080/13814788.2020.1757312

**Published:** 2020-04-30

**Authors:** An de Sutter, Carl Llor, Manfred Maier, Christian Mallen, Athina Tatsioni, Henk van Weert, Adam Windak, Jelle Stoffers

**Affiliations:** Department of Family Medicine, Care and Public Health Research Institute (CAPHRI), Maastricht University, Maastricht, The Netherlands

The novel coronavirus epidemic is transforming the world in which we live. This pandemic will bring sweeping changes everywhere, not least in the field of primary care medicine. Like one of our colleagues said: ‘after this crisis, perhaps even our calendar needs to be redefined. From now on, “BC” might stand for “Before Coronavirus”’. This quote puts into perspective just how significant the current times are for our profession. In this editorial, we will discuss challenges and tasks the COVID-19 crisis presents for family medicine.

## Challenges

The coronavirus crisis highlights a number of inherent weaknesses in our existing healthcare system. First, there are problems with accessing the essential resources needed to tackle this pandemic, with a lack of respirators, protective equipment and, above all, people. Clinical staff are becoming infected following contact with sick patients resulting in periods of quarantine; and some clinical staff pay the highest possible price – the loss of their life.

Aside from shortages of equipment and personnel, we have to deal with the emotional problems associated with this crisis. Everyone, though to varying degrees, suffers from stress, which spreads almost as quickly as the pandemic itself. Social media play a powerful role in communicating feelings of uncertainty or even panic. Insecurity, loneliness, distress, fear for one’s loved ones, lack of support and self-reliance spread across the message boards. The emotional impact of this pandemic on the public is unparalleled.

Family doctors also are dealing with new forms of stress. Many of us feel ill at ease with the new ways of working. As generalists, we are used to assessing health complaints based on our knowledge of the patient as well as our observations: what we see and hear is vital. Non-verbal communication, as well as physical examination, are essential tools to our practice. Now, however, we need to make decisions without seeing the patient and sometimes even without knowing him or her. The answer to a question like ‘Are these chest complaints due to COVID-19 or could it be heart failure?’, now needs to be answered by means of a mere phone call. The care of patients suffering the burden of chronic disease is transformed from a holistic, multi-professional service into merely providing prescriptions and making the occasional phone call. Even if we – and our patients – have the capacity to make video calls, it is difficult to assess whether a COPD patient’s condition is exacerbating, whether the diabetes still is under control, or if the medication we started a few days ago is effective and is well tolerated. Home visits are avoided as much as possible. When family doctors do see a patient, we need to protect ourselves with masks, goggles and a protective coverall, and we can only touch patients while wearing gloves. Greeting a patient with a warm handshake seems to belong to an era long past. This detached way of seeing patients seems vastly unsuited to a profession in which the doctor’s relationship of trust with the patient is crucial.

The uncertainty of the situation is heightened because we still know very little about the disease caused by the SARS-CoV-2 virus. The exact consequences for people’s health and wellbeing, although sometimes dramatic, are still challenging to quantify. As family doctors, we are unable to distinguish accurately clinically between those with a common cold or bronchitis, and those with a case of COVID-19. We cannot yet predict who will experience a mild respiratory infection and who will become a candidate for the intensive care unit. We can only suspect what the future complications will be for those who have survived a severe infection or a three-week stay at the intensive care unit. Finally, we have no idea how long this pandemic and the economic and social crisis accompanying it will last and it is unknown yet what toll it will take, globally. Will life ever get back to normal?

Amidst all of this uncertainty, the cooperation between countries seems to be dwindling. The pandemic has made many states not only ‘locked down’ but also closed off; not just physically – which is, under these circumstances, advisable – but also emotionally. In a time where it is vital to come together, to support each other, and to share information and ideas, the distance instead seems to be ever growing.

All of the preceding implies some serious threats for our future. The quarantine of people at elevated risk of developing the disease is now standard. Almost everywhere, schools, universities, concert halls, theatres, cinemas, shops, pubs, restaurants and all other places where people used to come together are closed. Many people find themselves in isolation at home with their family – or worse still – all alone; working remotely, taking care of their children, or watching Netflix. Many are lonely, afraid or under stress. Mental problems may well become a ‘secondary’ epidemic, posing new challenges.

On top of the emotional strain encountered from this pandemic, more direct and quantifiable consequences are already being foreseen. We know that this novel coronavirus will be the source of a catastrophic social and economic crisis – the most disruptive series of events since World War II. Each one of us will suffer the consequences and experience the aftermath.

Finally, there are also valid concerns being raised about the training of new medical professionals. Medical schools may not be officially closed, but clerkships and clinical rotations are postponed ‘until further notice’. Learning in the workplace is being replaced by online long-distance teaching. Despite the efforts of many medical teachers, this situation cannot go on for long without reducing the quality of the medical education of future doctors.

## Adaptation

Yet many of us do not experience the current situation as purely negative. While this crisis has revealed some of our weaknesses and places high demands on our ability to deal with its discomforts, it has also identified some of our strengths.

During these trying times, people have come together in different ways. Families are spending more quality time together, and neighbours are doing their best to take care of each other, offering help with the shopping and urgent repairs in other’s homes. This may have a lasting positive impact on social relations after the crisis, promoting community cohesion and building strong neighbourhoods.

Social media are being used not only to share emotions, but also to mobilise people to take action. They send words of encouragement, support and hope. They give tips on how to deal with specific problems. They provide knowledge and examples of the best possible practices and share latest evidence. They give a sense of belonging and unity in times of misery.

While individual citizens are taking positive action, many governments are also stepping up to the challenge. Although their decisions may vary in detail, they all have taken similar measures to reduce, slow down, and - hopefully - ultimately wholly stop the spread of the virus. In many countries, the measures taken were based on the best evidence provided by experts: epidemiologists, virologists, statisticians, physicians and others. Some of us wondered where the generalists’ voice was.

In healthcare itself, a revolution is taking place. Hospitals are reorganising at an impressive pace by creating new wards for infectious diseases and intensive care. National coordination centres are being established; regional cross-border collaboration developed spontaneously. In family medicine as well, massive changes are being made. Family doctors have reorganised their practices overnight to create separate patient streams of potential COVID patients from non-COVID patients, respectively. ‘Physical distancing’ in family practice is operationalised by a variety of measures, such as triage, phone or teleconsultation, removing chairs from waiting rooms and putting up Plexiglas screens at the reception, making it possible to keep the practice open and to welcome the patients who need seeing us. In many countries, local GP collectives – in ordinary times mainly active in organising out-of-hours services – joined forces with other primary care and welfare organisations to set up the care needed by the population during this epidemic. In no time they have developed procedures and protocols, set up distribution points for protective equipment, installed triage centres, and managed ‘corona centres’ – for patients not ill enough for the hospital but too ill to stay at home.

At the same time, new guidelines were developed by societies for family medicine and by professional expert groups at a speed unthinkable in normal times. Topics were – for example – protective measures, privacy-proof video call systems, triage of respiratory symptoms, shared decision-making on hospitalisation or referral to an intensive care unit, palliative care guidance, end-of-life decisions in frail patients with COVID symptoms, and many other issues.

## Tasks

Overall, this pandemic has genuinely demonstrated the medical professional’s power to adapt, evolve and thrive, even in these times of unprecedented crisis. Aside from the enormous challenges this novel coronavirus poses – and will continue to pose for a long time to come – it also presents us with new tasks. The crisis has put family medicine at the centre of the health care system, even among countries with a health care system that traditionally has been hospital centred, and has highlighted its strengths: patients count on their family doctor for information, advice, reassurance, forward referral, and all the other critical components of primary care more than ever. For hospital medicine, meanwhile, our gatekeeping role is indispensable to control not only the influx of patients but also the spread of COVID-19 among visiting patients or hospital staff.

Family doctors can – and therefore must – play a crucial role in COVID-19 research through the careful registration of all their COVID-19 patients; both survivors and the deceased, including patients who tested positive for a virus test, as well as patients with typical COVID-like symptoms who were not tested – and most often were not hospitalised – but recovered at home, in isolation. This is essential to obtain reliable data on the ‘true’ point prevalence, cumulative incidence and mortality of COVID-19. Furthermore, in the near future valid serological tests will become available. Thus, we then can determine in representative population samples – consisting of persons with or without symptoms, with or without contact with a healthcare service – who has had COVID-19 and who has not. By combining outcomes – SARS-CoV-2 immune status and mortality from COVID-19 – with data extracted from hospital and primary care information systems, informative databases can be created. Family doctors, hospital specialists, virologists, public health experts, epidemiologists, and data scientists should join forces to analyse these datasets. Our primary care information systems contain a wealth of clinical data – i.e. recordings of basic physical characteristics (age, sex, height, weight), lifestyle factors, symptoms, signs, laboratory test results, comorbid conditions, and medication – which may be associated with COVID-19. We need the results – disease occurrence, risk factors and protective factors, diagnostic models, models predicting a favourable or severe outcome, and options for intervention and prevention – to fight the next wave of COVID-19; and to determine who needs to be vaccinated first. Developing standardised data-reporting protocols for primary health care among European countries may provide robust information for future research, considering regional differences in spread of the virus and measures taken. Finally, primary health care should not spare efforts that continue to demonstrate its effectiveness, safety and patient centredness in the era of COVID-19. For example, on one end of the primary care spectrum, multicentre clinical trials might evaluate the telemedicine solutions commonly used during the coronavirus crisis, and on the other end, integrated care models including home visits, could be evaluated.

## Conclusion

All of the above highlights how the COVID-19 epidemic presents demanding challenges for family medicine. Family doctors and their staff work in the frontline of the epidemic. They must be able (and otherwise be enabled) to protect themselves – and their patients – and stay safe. Family medicine, dealing with both the emotional and the scientific side of medicine on a daily basis, plays a central role in tackling this pandemic. Taking responsibility for both care and research on COVID-19 will redefine the importance of family medicine for public health care. That is the generalists’ voice.

